# Navigating Compound Odontomas: A Case Study of Surgical Intervention and Full-Mouth Rehabilitation in a Pediatric Patient

**DOI:** 10.7759/cureus.73116

**Published:** 2024-11-06

**Authors:** Thikra O Alsharif, Yasser Ibrahim, Afnan S Aljohani

**Affiliations:** 1 General Dentistry, Umm Al-Qura University, Mecca, SAU; 2 Maxillofacial Surgery, Security Forces Hospital, Mecca, SAU; 3 Pediatric Dentistry, Security Forces Hospital, Mecca, SAU

**Keywords:** compound odontoma, dental caries, full mouth rehabilitation, odontogenic tumor, pediatric pathology

## Abstract

Odontomas are the most prevalent odontogenic tumors, often classified as hamartomas due to their slow growth and non-aggressive nature. Typically asymptomatic, they can obstruct the eruption of adjacent teeth. While the exact causes of odontomas remain unclear, potential factors include local trauma, infection, growth pressure, and hereditary influences. Radiographically, they appear as well-defined radiopaque lesions surrounded by a radiolucent halo and are categorized into complex and compound types based on the arrangement of dental tissues.

This case report aims to document the surgical removal of a compound odontoma in the lower right jaw of an 8-year-old girl, which obstructed the eruption of her lateral incisor, along with the subsequent full-mouth rehabilitation performed under general anesthesia. Diagnostic evaluations included oral and radiographic examinations such as orthopantomograms (OPG), periapical (PA), bitewing (BW), and computed tomography (CT) scans. The patient had no medical or family history of dental anomalies.

This report highlights the management of a compound odontoma causing delayed eruption and impaction and demonstrates the effectiveness of full-mouth rehabilitation under general anesthesia in a pediatric setting. Early detection and removal of odontomas are crucial to preventing eruption issues and long-term complications, although recurrence may necessitate ongoing observation. The findings underscore the importance of early diagnosis and intervention in managing odontomas to mitigate adverse effects.

## Introduction

Odontomas are benign developmental malformations of odontogenic origin and often appear as small, solitary, or multiple radiopaque lesions discovered during routine radiographic exams [1]. They represent a significant proportion of odontogenic tumors and are the most common lesions reported in studies. In an analysis by Buchner et al. (2006) of 1,088 odontogenic tumors, odontomas accounted for 75.9% of cases [2,3]. Their slow growth and non-aggressive nature support their classification as hamartomas rather than true neoplasms, as they consist of normal-appearing epithelial and mesenchymal cells without proper structural arrangement [4,5].

The term "odontoma" was first introduced by Broca in 1866, who defined it as a tumor formed by the overgrowth of complete dental tissue [6]. Odontomas are typically asymptomatic but can sometimes interfere with the eruption of associated teeth, leading to impaction or delayed eruption [7]. The exact cause of odontomas remains unclear, though factors such as local trauma, infection, growth pressure, and hereditary or developmental influences have been suggested as possible contributors [8].

The fifth edition of the World Health Organization’s Classification of Odontogenic Tumors, which opened to online access in March 2022, classifies odontomas into two main types: complex and compound. Complex odontomas consist of well-formed dental tissues arranged in an amorphous and somewhat disorganized manner. In contrast, compound odontomas exhibit dental tissues arranged in an orderly pattern but with altered size and conformation, leading to the formation of small tooth-like elements known as odontoids or denticles. Additionally, a mixed form, incorporating both compound and complex characteristics, can also be found [9,10].

Radiographically, odontomas are highly distinctive. They typically appear as well-defined radiopaque lesions surrounded by a radiolucent halo, representing an enlarged cystic follicle. Compound odontomas demonstrate radiopaque areas with opacities similar to those of normal teeth. In contrast, complex odontomas present as a radiopaque solid mass with occasional nodular components. Both types of odontomas are classified as unilocular lesions, clearly separated from normal bone by a well-defined line of corticalization [2,11].

Odontomas are detected during routine radiographic examinations [12]. Compound odontomas are more commonly found in the anterior region of the jaw, while complex odontomas are more prevalent in the posterior region. Interestingly, both types tend to occur more frequently on the right side of the jaw than on the left [13].

Surgical removal is the standard treatment for odontomas, with recurrence being rare [14]. Surgery is usually performed to prevent complications with permanent tooth eruption, particularly in pediatric patients [15]. Differential diagnosis should consider other ossified bone lesions, such as ossifying fibroma, odontoameloblastoma, ameloblastic fibroma or fibro-odontoma, osteoma, fibrous dysplasia, or florid osseous dysplasia [16].

The aim of this case report is to document the surgical removal of a compound odontoma that may obstruct the eruption of the lateral incisor, along with full mouth rehabilitation performed under general anesthesia. This report also highlights the treatment approach and outcomes in a medically healthy patient with no history of trauma or family history of dental anomalies.

## Case presentation

**Figure 1:**
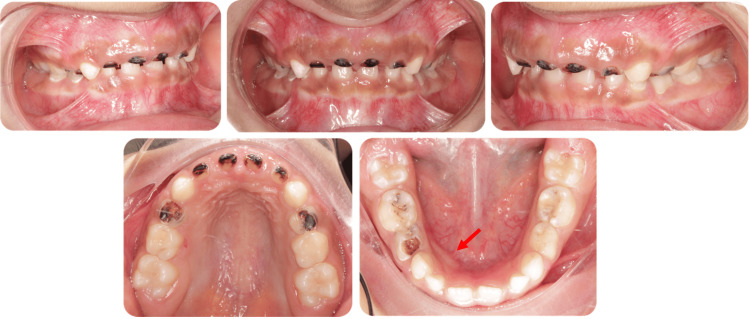
Pre-operative intraoral photographs Show multiple carious lesions across various surfaces, with the red arrow indicating the odontoma location.

An eight-year-old Saudi girl with no significant medical history, no known drug allergies, no known food allergies, and no history of trauma or family dental anomalies presented to the Security Forces Hospital in Makkah, Saudi Arabia, seeking dental treatment for multiple carious teeth. Extraoral examination was within normal limits (WNLs), revealing a mesofacial pattern with normal facial symmetry and a slightly convex facial profile. Intraoral examination (Figure 1) showed soft tissues with WNLs and hard tissues with multiple carious lesions, as well as a mild palpable swelling on the lingual aspect of the lower right side.

**Figure 2:**
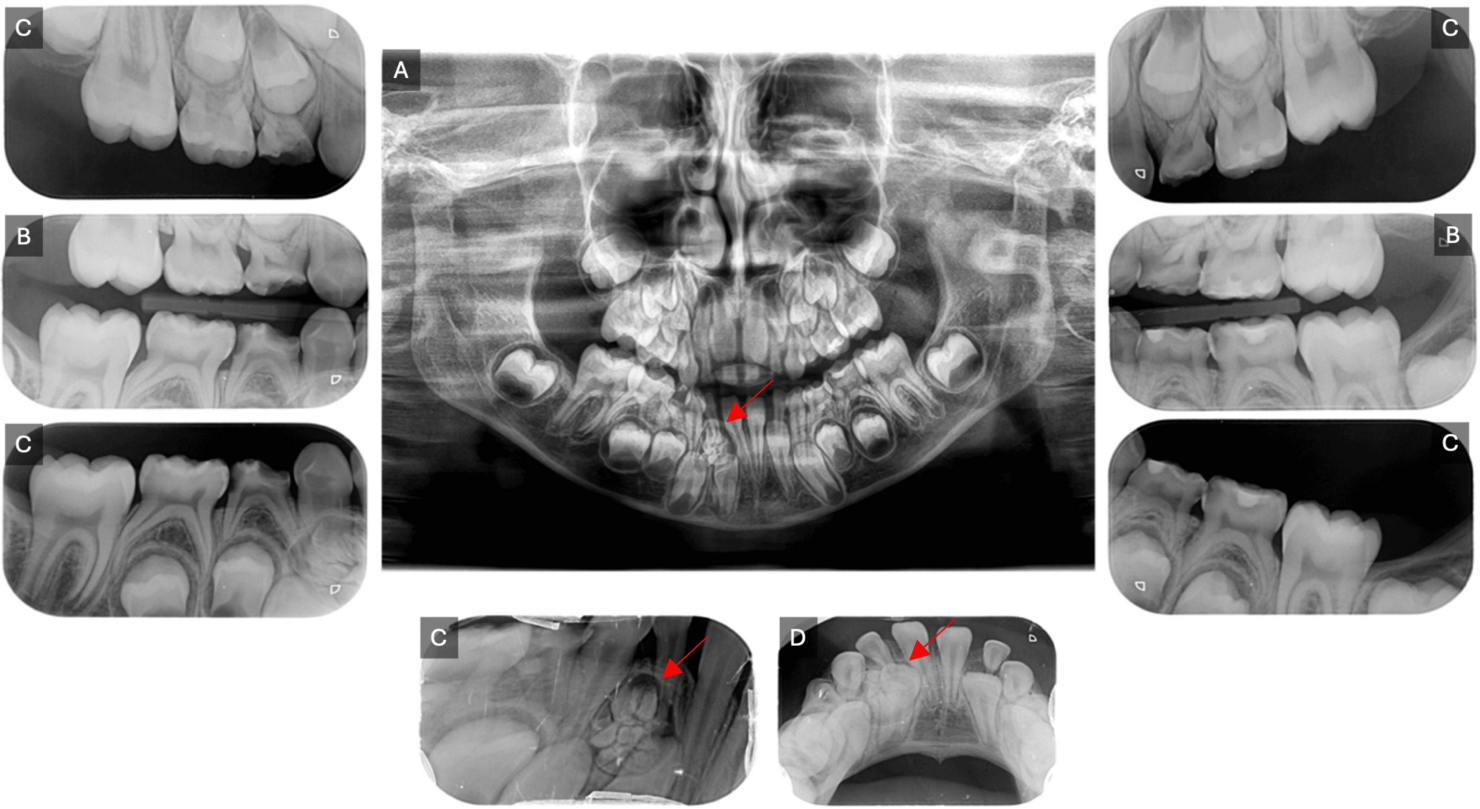
Pre-operative radiographs (A) a panoramic view (OPG) revealing a possible obstruction of the right mandibular permanent lateral incisor's eruption by an odontoma compared to the left side; (B) a bitewing radiograph displaying multiple carious lesions and highlighting the extent of caries; (C) a periapical radiograph, and (D) an occlusal radiograph, with red arrows indicating areas of interest, demonstrating a small calcified structure resembling tooth-like formations.

**Figure 3:**
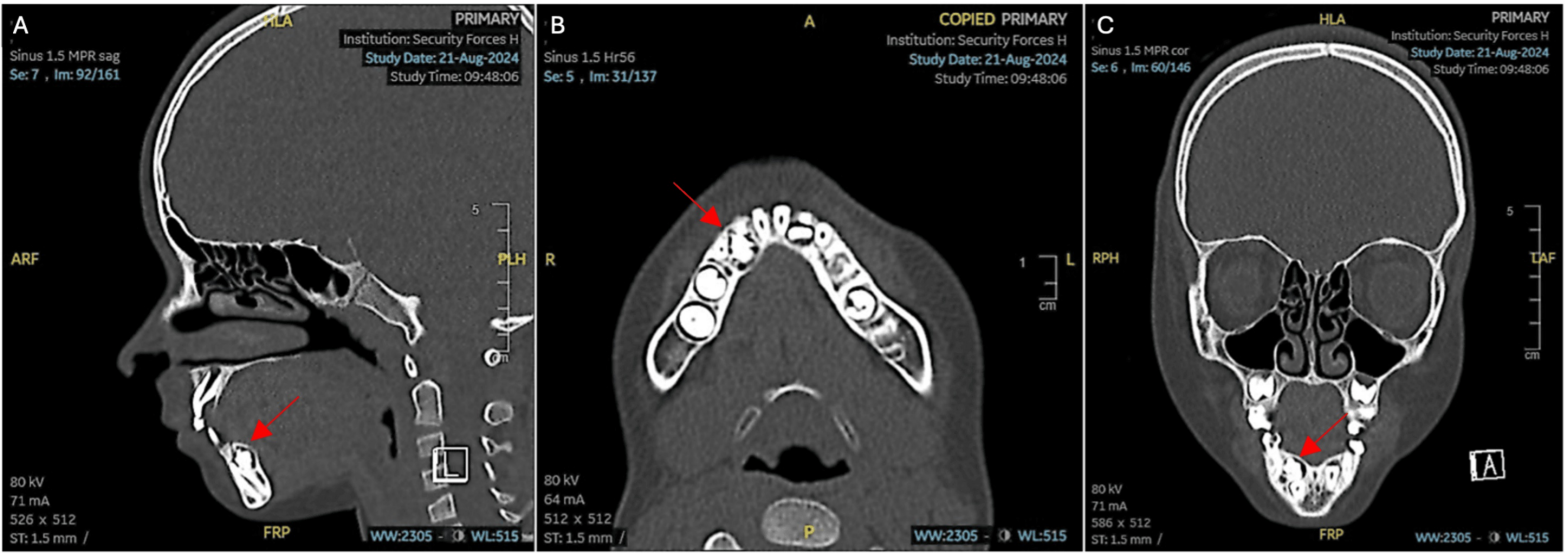
Pre-operative computed tomography (CT) scan (A) Sagittal section, (B) Axial section, (C) Coronal section, illustrating the location of the odontoma between the primary and permanent lateral incisors, extending more toward the lingual bone plate.

Routine radiographic examination (Figure 2) (OPG, PAs, BWs) identified multiple carious lesions and a radiopaque mass consisting of small, calcified structures resembling normal teeth, surrounded by a narrow radiolucent zone that might obstruct the eruption of the right mandibular permanent lateral incisor compared to the left side. A consultation with oral and maxillofacial surgery (OMFS) was conducted, after which a CT scan was performed to determine the exact location of the odontoma and to plan the surgical approach (Figure 3). The patient underwent full-mouth rehabilitation and odontoma removal under general anesthesia, with written informed consent obtained from her guardian.

Preoperative investigations included routine blood tests and a pre-anesthetic evaluation conducted by the anesthesiology department to clear the patient for treatment under general anesthesia. The dental team planned to first perform the odontoma removal procedure, followed by comprehensive dental treatment.

Odontoma removal

**Figure 4:**
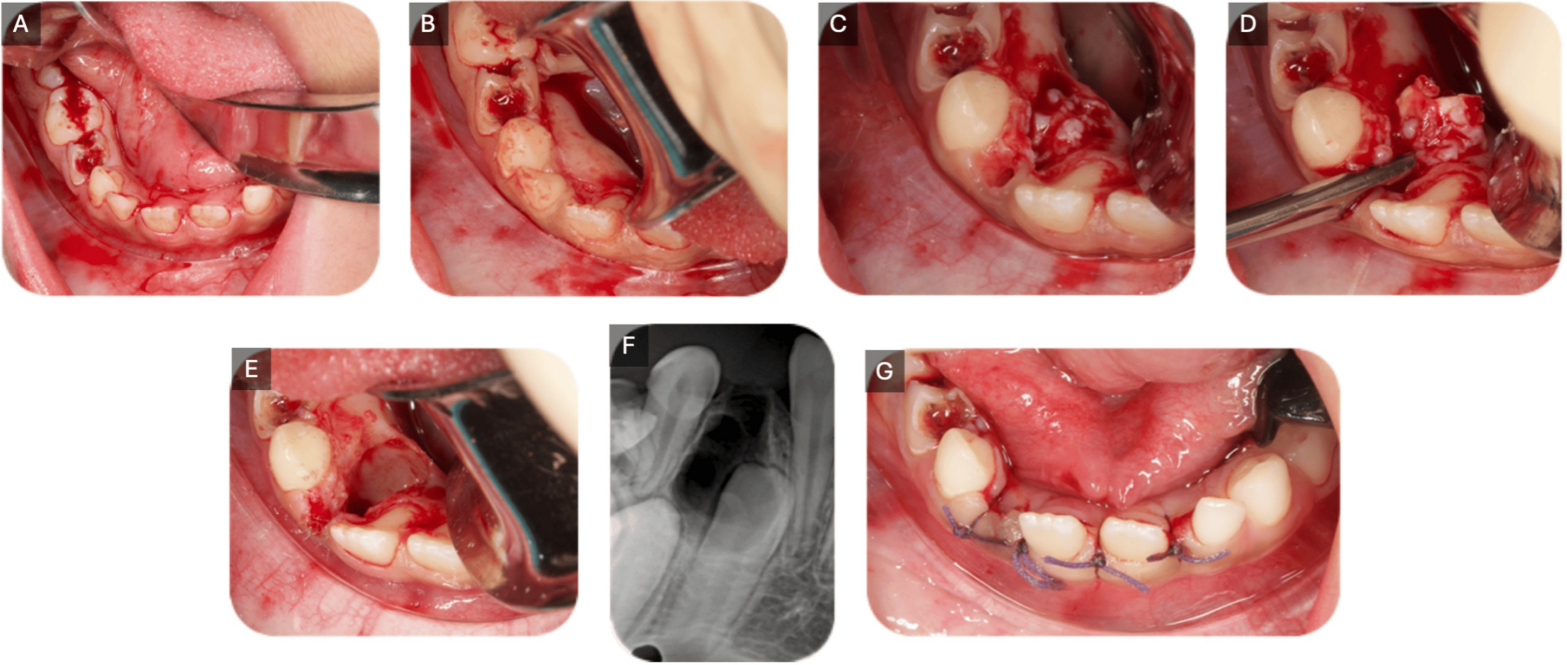
Surgical excision of the odontoma (A) preoperative site, (B) reflection of the mucoperiosteal flap, (C) extraction of the right mandibular primary lateral incisor and creation of a bone window, (D) removal of the odontoma, (E) complete removal of the compound odontoma, (F) postoperative intraoral periapical radiograph taken prior to suturing to confirm complete removal, and (G) wound closure with 3-0 silk sutures.

**Figure 5:**
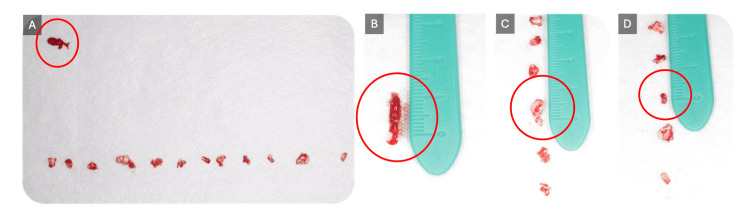
The excised odontogenic lesion (A) 12 enucleated denticles with a fibrous capsule; (B) the diameter of the fibrous capsule; (C) the largest denticle; and (D) the smallest denticle.

After raising a lingual mucoperiosteal flap and extracting the right mandibular primary lateral incisor, a bone window was created to access the odontoma (Figure 4). The surgical removal was carried out by the maxillofacial oral surgery team, with close attention to preserving the integrity of the lingual nerve and minimizing any risk of damage. Additionally, care was taken to avoid disrupting the follicle of the underlying permanent teeth and neighboring structures, ensuring minimal impact on surrounding dentition and supporting tissues. The excised tumor consisted of 12 denticles of various sizes and shapes along with the follicle (Figure 5).

Comprehensive dental treatment

The treatment plan carried out by the pediatric dentistry team included applying fissure sealants, performing conservative adhesive restorations, and placing light-cure composite restorations. Pulpotomies were performed, and stainless-steel crowns were placed. Extractions were also conducted on several teeth, as indicated.

**Figure 6:**
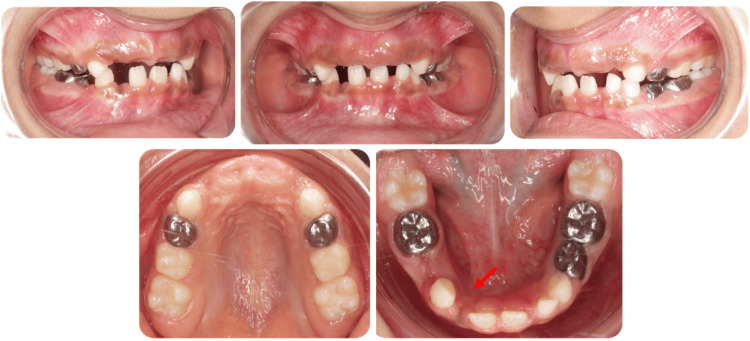
Postoperative photographs illustrate the application of pit and fissure sealants to the maxillary first permanent molars, conservative adhesive restorations in the maxillary second primary molars, light-cure composite restorations in the right mandibular primary canine, and stainless-steel crowns in the maxillary first primary molars, the left mandibular primary molars, and the right mandibular second primary molar. Extractions were performed on the maxillary primary incisors, the right mandibular first primary molar, and the right mandibular primary lateral incisor to facilitate access to the surgical site, with the red arrow indicating the healing site of the odontoma surgical area.

**Figure 7:**
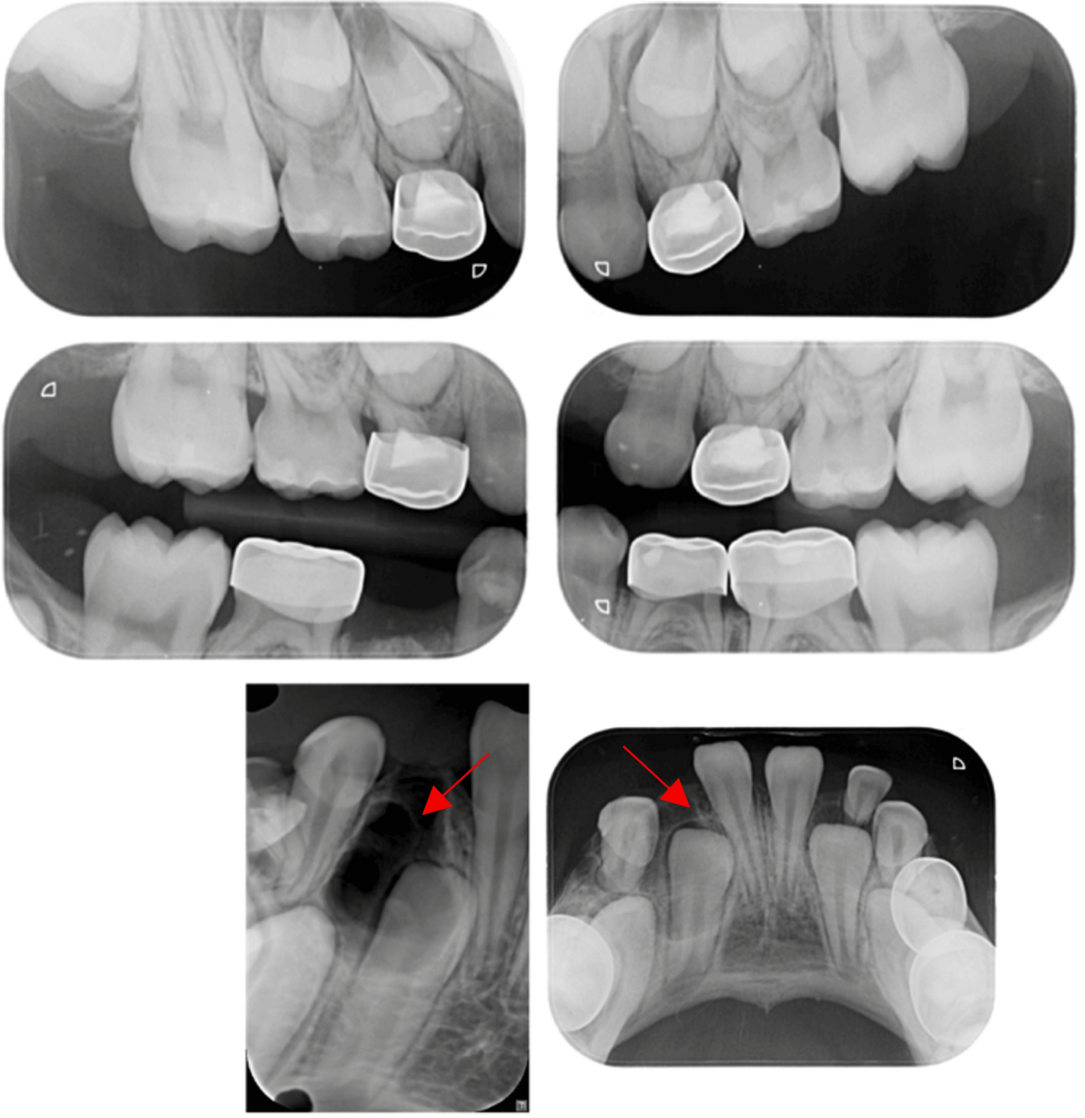
Postoperative radiographs Show the pulpotomies in the maxillary first primary molars, the placement of stainless-steel crowns, the extracted sites, and confirm the complete removal of the odontoma at the surgical area.

Postoperative radiographs and photographs were taken (Figures 6, 7). The patient's guardian received home-care instructions, including oral hygiene and dietary counseling. A follow-up protocol was implemented, which included a one-week postoperative visit to monitor initial healing, followed by a two-week visit for suture removal. The schedule then consisted of assessments at three months and six months in the first year, focusing on stabilizing the patient's oral hygiene and monitoring the eruption of the right mandibular permanent lateral incisor. After the first year, annual recall visits were implemented to monitor for the recurrence of the odontoma, assess the patient’s oral hygiene and caries risk, ensure the long-term success of the surgical intervention and rehabilitation, and perform any necessary dental procedures. This annual follow-up will continue throughout the patient’s life to maintain optimal oral health.

**Figure 8:**
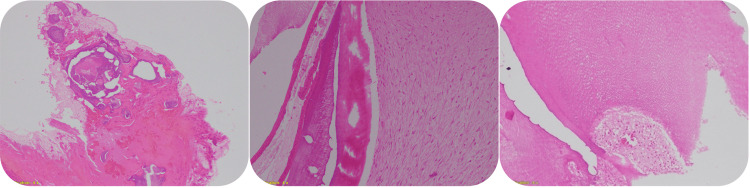
Histopathological examination The examined sections revealed dental hard tissues, including enamel matrix, mineralized dentin, and dental pulp, along with mesenchymal components embedded within dense fibrous tissue, formed by follicular epithelium.

Histopathological examination of the denticles confirmed the diagnosis of a compound odontoma (Figure 8).

## Discussion

Odontomas are benign tumors of odontogenic origin and are recognized as the most prevalent type of odontogenic tumor [1,2]. Generally, odontomas are asymptomatic; they can occasionally cause bone expansion and are associated with impacted or unerupted teeth in approximately 80% of instances [10]. Clinical manifestations may include eruption disturbances, cortical bone expansion, misalignment of teeth, and pain [10]. This is similar to our case, where the odontoma was asymptomatic and discovered during a routine radiographic examination, obstructing the eruption of the right permanent mandibular lateral incisor and positioning it lower than the left, as shown in OPG (Figure 2).

Diagnosis typically relies on routine radiological examinations, such as panoramic and intraoral radiographs, particularly when delayed tooth eruption is present. While radiographic assessments effectively distinguish different odontoma types, a definitive diagnosis requires histopathological examination [17]. In the current case, an intraoral radiograph revealed multiple tooth-like structures beneath the root of the deciduous tooth and above the crown of the unerupted permanent lateral incisor. A CT scan was then performed to determine the exact position of the odontoma and plan the surgical approach. The initial diagnosis of compound odontomas was based on these findings, and surgical removal was recommended. The enucleated specimen was sent for histopathological analysis, which confirmed the diagnosis and helped differentiate the odontoma from similar lesions, such as ameloblastic fibro-odontomas and odontoameloblastomas, which can closely resemble odontomas radiographically.

According to Kaban, odontomas are generally easy to remove surgically, as they can usually be enucleated without damaging adjacent teeth. This is often because a thin bony septum separates the odontoma from neighboring teeth, protecting them during removal. While odontomas have limited growth potential, it’s still recommended that they be removed. This is due to the presence of various tooth-like structures within odontoma, which can increase the risk of cyst formation over time [18]. A conservative surgical approach is typically effective, with a low risk of recurrence. Early detection and timely removal help prevent complications like tooth loss, cyst formation, bone expansion, and delayed eruption of permanent teeth [10]. In the current case, the odontoma was surgically removed to address its obstruction of the permanent tooth's eruption pathway and to prevent potential complications such as cyst formation or delayed eruption, which align with findings in existing literature. As supported by Kaban’s study, early detection and removal of odontomas are essential to preventing complications and promoting normal dental development [18]. The conservative approach that has been followed in the current case, involving enucleation of the lesion and extraction of the primary tooth, aligns with established practice by providing a clear path for the permanent tooth to erupt.

In pediatric patients, where the bone remodeling rate is three times higher than in adults [19], placing a bone graft at the tumor site is unnecessary in the current case. We followed this approach, deciding not to place a bone graft, as rapid natural bone healing was anticipated in our young patient. While recurrence after enucleation is rare, close monitoring in young children is essential. Additionally, implementing preventive and interceptive orthodontics, if needed, can help avoid future malocclusion [10]. Following recommendations from previous research, we emphasized close monitoring post-surgery to detect any signs of recurrence and utilized orthodontic measures to minimize the risk of future alignment issues.

## Conclusions

This case report presents the surgical removal of a compound odontoma that obstructed the eruption of a mandibular right permanent lateral incisor in a pediatric patient. Following the procedure, the patient underwent comprehensive dental rehabilitation under general anesthesia. Currently, the tooth has not erupted, and the patient is under follow-up to monitor progress. Early detection and removal of odontomas are essential to prevent eruption issues and long-term complications, though recurrence may require ongoing observation. The report highlights the value of early diagnosis and intervention in managing odontomas and preventing adverse effects.

## References

[1] Neville BW, Damm DD, Allen CM, Chi AC (2015). Oral and maxillofacial pathology (e-book). https://books.google.co.in/books?hl=en&lr=&id=DmVgDwAAQBAJ&oi=fnd&pg=PP1&dq=Oral+and+maxillofacial+pathology+(e-book)&ots=9HZQ9G482e&sig=zheaBmSpZRXYbhme0n-O1E1uOi0&redir_esc=y#v=onepage&q=Oral%20and%20maxillofacial%20pathology%20(e-book)&f=false.

[2] Hidalgo-Sánchez O, Leco-Berrocal MI, Martínez-González JM (2008). Metaanalysis of the epidemiology and clinical manifestations of odontomas. Med Oral Patol Oral Cir Bucal.

[3] Buchner A, Merrell PW, Carpenter WM (2006). Relative frequency of central odontogenic tumors: a study of 1,088 cases from Northern California and comparison to studies from other parts of the world. J Oral Maxillofac Surg.

[4] Shafer WG, Hine MK, Levy BM (1963). A textbook of oral pathology. https://www.researchgate.net/publication/286398753_Shafers_textbook_of_Oral_pathology.

[5] Shekar S, Rao RS, Gunasheela B, Supriya N (2009). Erupted compound odontome. J Oral Maxillofac Pathol.

[6] Cohen DM, Bhattacharyya I (2004). Ameloblastic fibroma, ameloblastic fibro-odontoma, and odontoma. Oral Maxillofac Surg Clin North Am.

[7] Gervasoni C, Tronchet A, Spotti S (2017). Odontomas: review of the literature and case reports. J Biol Regul Homeost Agents.

[8] Hitchin AD (1971). The aetiology of the calcified composite odontomes. Br Dent J.

[9] Soluk-Tekkesin M, Wright JM (2022). The World Health Organization classification of odontogenic lesions: a summary of the changes of the 2022 (5th) edition. Turk Patoloji Derg.

[10] Zidane FE, Azzouz Y, Fawzi R (2022). Surgical management of compound odontoma associated with unerupted tooth: a case report. Pan Afr Med J.

[11] Lee G, Singer SR (2010). Concomitant occurrence of periapical cemental dysplasia and compound odontoma in the anterior mandible: report of a case. J Orofac Sci.

[12] Snawder KD (1974). Delayed eruption of the anterior primary teeth and their management: report of a case. ASDC J Dent Child.

[13] Kannan KS, Prabhakar R, Saravanan R, Karthikeyan Karthikeyan, Rajvikram Rajvikram (2013). Composite compound odontoma-a case report. J Clin Diagn Res.

[14] Ćabov T, Fuchs PN, Zulijani A, Ćabov Ercegović L, Marelić S (2021). Odontomas: pediatric case report and review of the literature. Acta Clin Croat.

[15] Altay MA, Ozgur B, Cehreli ZC (2016). Management of a compound odontoma in the primary dentition. J Dent Child.

[16] Park JC, Yang JH, Jo SY, Kim BC, Lee J, Lee W (2018). Giant complex odontoma in the posterior mandible: A case report and literature review. Imaging Sci Dent.

[17] Abdul M, Pragati K, Yusuf C (2014). Compound composite odontoma and its management. Case Rep Dent.

[18] Kaban LB, Troulis MJ (2004140). Pediatric oral and maxillofacial surgery.

[19] Parfitt AM, Travers R, Rauch F (2000). Structural and cellular changes during bone growth in healthy children. Bone.

